# State Registration and Its Values

**Published:** 1920-08-28

**Authors:** 


### THE MATRONS' AND SISTERS' DEPARTMENT.

### STATE REGISTRATION AND ITS VALUES.

### I. The New Factor in the Nurse's Life.

As the days of this disappointing summer pass swiftly away, the time grows ever nearer which will introduce into the lot of every nurse in the kingdom an untried condition. Very soon now the preliminaries will be settled, and the General Nursing Council will announce the opening of the office for State registration. Every nurse will then have before her the necessity for making a decision on a matter which affects her very nearly indeed. Shall she apply to be registered? Shall she not? When it comes to the point we cannot but foresee that there will be many waverers, many refusing to see "the good of it," many, maybe, so careless and inattentive that they have hardly ever heard of State registration, much less taken in all its pros and cons.

Truth to tell State registration does not in this, its first appeal, come with a very strong case before the best-trained members of the profession. For when they ask "What is the qualification for becoming a State registered nurse?" they learn that any woman who bears a good character, is of the prescribed age, and can show that she has been in *bonâ-fide* practice as a nurse in attendance on the sick during the last three years under conditions approved by the Council, is to be eligible, and this for a period of two years to come. Now this is a very hard pill to swallow. It means that for a long term of years, practically during the lifetime of this generation of registered nurses, they will find themselves in the same category with people who, though they may be of excellent character, though they may have done a considerable amount of nursing, are yet not fully-trained according to the modern signification of the term. We know how easily people slipped into nursing posts at one time during the war and before it. Women who passed a year or two at a little cottage hospital, or took a course of training at a hospital receiving but one kind of patient, women who had been in women's wards but had never nursed a man, and *vice versa*, people who had been "trained" in nursing homes—these, it would seem by the terms of the Registration Bill, if qualified by some years of *bonâ-fide* practice as nurses, must be allowed to take their place among the ranks of the registered. "Is it good enough?" the trained nurse is beginning to ask as she realises these things. That some will decide in the negative is already a foregone conclusion. Very many are inquiring how, when they are once registered, they can be distinguished from the *bonâ-fide* nurses.

It must be frankly confessed that there is no way in which the State Register can distinguish the trained from the untrained except by the entries made in the register. It neither could nor should make a difference between the two classes on whom it confers its cachet. But a reference to its register will, of course, at once disclose whether the nurse has had adequate training or not. For a long time to come registration will not act as a hall-mark of training. It can only register the certificates of those it accepts. But year by year the number of the registered who are not, strictly speaking, trained will grow less, and the proportion of well-certified nurses will increase, until at length registration will unite the profession and confer all that it desires. Long before that time is reached it may be safely prophesied that no nurse entitled to enter will venture to remain outside the fold.

The business of admitting to the register all who can prove they have been following the profession of nursing with credit, irrespective of their qualifications, must last two years. It is of the utmost importance for every individual nurse to make up her mind during this time to apply for registration. At the end of the term prescribed by law entry to the register will be considerably more difficult. It is proposed to admit by examination, and candidates will have to qualify for entry by a stated term passed in hospitals. We know of no man or woman who would choose to be examined even on the subjects he knows best, if the desired status could be had without this ordeal. So nurses should remember if they sit on the fence, inattentive to their own interests a day too long, if they stake their professional career on the chance that registration is not going to be any good to them, and discover that without it they are the object of general suspicion, then there will be nothing for it but to get coached once more in all the old subjects and present themselves for examination. It is rather a horrid risk to run for the sake of a guinea and a letter or two.

Of course, there is one very easy way of getting distinguished from the *bonâ-fide* nurses on the Register. Nurses who can satisfy the Registration Board of the College of Nursing will find themselves possessed of a hall-mark none can dispute. With the College badge to serve as an easily recognisable sign of their modernity they need not feel annoyed if they find their name is wedged in between two *bonâ-fide* nurses in the State Register.

(*To be continued.*)

